# Effect of Heat Treatment Condition on the Flow Behavior and Recrystallization Mechanisms of Aluminum Alloy 7055

**DOI:** 10.3390/ma12020311

**Published:** 2019-01-20

**Authors:** Bin Liao, Lingfei Cao, Xiaodong Wu, Yan Zou, Guangjie Huang, Paul A. Rometsch, Malcolm J. Couper, Qing Liu

**Affiliations:** 1 International Joint Laboratory for Light Alloys (Ministry of Education), College of Materials Science and Engineering, Chongqing University, Chongqing 400044, China; bin.liao@foxmail.com (B.L.); yanzou@cqu.edu.cn (Y.Z.); gjhuang@cqu.edu.cn (G.H.); qingliu@cqu.edu.cn (Q.L.); 2 Electron Microscopy Center of Chongqing University, Chongqing University, Chongqing 400044, China; 3 Department of Materials Science and Engineering, Monash University, Clayton, VIC 3800, Australia; paul.rometsch@monash.edu (P.A.R.); malcolm.couper@monash.edu (M.J.C.)

**Keywords:** 7055, aluminum alloys, hot compression, flow stress decline ratio map, processing map, deformation softening, recrystallization

## Abstract

The flow behavior and the microstructural evolution of aluminum alloy 7055 in two heat treatment conditions (homogenized vs. pre-rolled, solution treated, stretched and naturally aged (T3)) were investigated for a height reduction of 60% with deformation temperatures ranging from 370 °C to 450 °C and strain rates ranging from 0.01 s^−1^ to 10 s^−1^. Flow stress decline ratio maps as a function of deformation temperature and strain rate were produced along with processing maps at a strain of 0.8 to reveal optimum hot-working conditions for deformation at strain rates of 0.01 s^−1^ to 0.1 s^−1^. The results showed that the stress drop ratio during deformation is higher for the homogenized condition than for the pre-rolled, T3 condition. A higher degree of recrystallization after deformation was observed in the pre-rolled, T3 condition due to finer second phase particles, smaller grain size, and more numerous sub-grains. The mechanism for deformation softening is discussed in the context of grain boundary characteristics.

## 1. Introduction

Al-Zn-Mg-Cu alloys have been a primary choice of structural materials for aircraft due to their low weight, high specific strength, excellent fracture toughness and good stress corrosion cracking resistance [1]. Alloy 7055, with its higher Zn/Mg ratio and lower Fe and Si contents, is an outstanding example of high-strength Al-Zn-Mg-Cu alloys, especially for application in compression-dominated structures. During the manufacture of 7055 thick plates, the DC-cast ingot is generally homogenized and hot rolled before solution treatment. Rolling aims to reduce the thickness of ingots, eliminate residual solidification porosity, reduce the size of intermetallics in the microstructure and refine grains to improve subsequent processing and mechanical properties. During hot rolling, dynamic recovery (DRV), and dynamic recrystallization (DRX) can happen, which are typical softening mechanisms in aluminum alloys at elevated temperature. DRV involves the annihilation and rearrangement of the dislocations; while DRX involves the development of high angle grain boundaries and/or the formation of new grains.

In order to understand the hot-formability and microstructural evolution of the alloy during hot deformation, thermal simulation technique has been utilized to monitor strain–stress behaviors, derive processing maps and develop constitutive models [2]. Thermal simulation studies on the hot-formability and microstructure of cast AA7055 alloys have been reported [3,4,5,6,7,8,9]. Yan [4] derived processing maps for deformation at temperatures from 300 °C to 450 °C to determine suitable processing windows for hot rolling, forging and extrusion, and discovered that flow instability is most likely to occur at high strain rates (>1 s^−1^). Constitutive equations and models have been established to predict flow stress behavior in alloy 7055 deformed at 300 °C to 450 °C with strain rates from 0.01 s^−1^ to 10 s^−1^ [4,8,9]. The microstructure was influenced by deformation parameters such as temperature, strain rate, reduction rate, and by the design of deformation passes [5,7]. Results from such work have demonstrated that the dislocation density decreases and the average sub-grain size increases with (i) increasing temperature, (ii) increasing number of deformation passes, (iii) decreasing strain rate and (iv) decreasing reduction rate. Electron back-scattered diffraction (EBSD) technique is widely used together with thermal simulation to understand deformation and recrystallization mechanism during hot deformation. Yan et al. [3,6] studied the recrystallization and recovery of hot deformed samples by EBSD and found that static recovery and meta-dynamic (i.e., post-deformation) recrystallization happened during the interval between two passes. Specially, grain orientation information [10] and the grain orientation spread (GOS) [11,12] data can be derived from EBSD results to identify the degree of deformation of the grains.

So far, most of the thermal-mechanical simulation work on alloy 7055 has been carried out on homogenized cast samples, whilst in practical production, the alloy can also be rolled to thick plates, solution treated, stretched, and then stored or shipped in the naturally aged condition (i.e., T3) prior to further rolling. This kind of treatment raises questions about whether there are any differences in flow stress behavior and recrystallization mechanisms for alloy 7055 for the rolling of homogenized material compared to the rolling of the pre-rolled (T3) condition, in the event that such a final forming step is required (or desirable). The answer is critical to understanding the workability of the alloy under different processing conditions and providing flexibility in the production design in manufacturing.

Therefore, in this study, the hot-formability of alloy 7055 was investigated at typical production deformation temperatures for both homogenized and pre-rolled (T3) samples on the basis of stress–strain curves and microstructure evolution. Flow stress decline ratio maps and processing maps were established to determine optimized deformation parameters and identify regions of instability for hot working. The findings are discussed in relation to the sub-structure, recovery, and recrystallization mechanisms.

## 2. Materials and Methods

### 2.1. Materials

The material used in this work was alloy 7055 (7.7 Zn, 2.2 Mg, 2.0 Cu, 0.12 Zr, 0.04 Fe, 0.03 Si, 0.04 Ti, 0.01 Cr, 0.01 Mn and the balance Al, in wt %), which was direct chill cast to approximate dimensions 4000 × 500 × 400 mm^3^, then homogenized at 460 °C for 6 h, followed by 465 °C for 24 h then water quenched. Half of the material was then hot rolled at 400 °C to a final thickness of 20 mm, solution treated at 475 °C for 3 h, quenched by water spray, stretched by 2% and naturally aged for more than 10,000 h at room temperature.

### 2.2. Hot Compression

Cylindrical compression samples, measuring 8 mm in diameter and 12 mm in height, were machined in the through thickness direction of the homogenized material and in the longitudinal direction of the rolled material. Standard uniaxial compression tests were performed on a Gleeble-3800 thermal simulation machine (3800D, Dynamic Systems Inc., Austin, TX, USA) over a temperature range of 370 °C to 450 °C with strain rates from 0.01 s^−1^ to 10 s^−1^. All samples were heated to the testing temperature at a heating rate of 2.5 °C/s and held for 3 min before compression at a fixed strain rate. The specimens were deformed to a height reduction of 60%, and then water quenched immediately to preserve the deformed microstructure.

### 2.3. Microstructure Characterization

The microstructures were characterized using a field emission scanning electron microscope (SEM, TESCAN MIRI 3, Brno, Czech Republic) with an electron backscatter diffraction (EBSD) detector (EBSD, EDAX Inc., Oxford Instruments, Abingdon, UK) and a backscattered electron (BSE, TESCAN, Brno, Czech Republic) detector. The EBSD specimens were sectioned parallel to the compression axis of the deformed specimens, and the examined surface was prepared by grinding (200# to 4000# emery paper) then electropolishing in a solution of 10% HClO_4_ and 90% C_2_H_5_OH at 20V. The EBSD maps were obtained by using an HKL technology EBSD system (version 3.2, EDAX Inc., Abingdon, UK) interfaced to a TESCAN MIRI 3, operated at 20 kV. Sub-grains were defined by a misorientation angle from 2–15°. Grain size was determined using the EBSD Channel 5 software with a critical misorientation for different grains of >15°. Misorientation gradients in each grain were estimated by GOS, which is defined as the misorientation between all measurement points of a grain and the grain average orientation. Partitioning was carried out by a GOS approach considering a 5° grain tolerance angle using the standard procedure of grain dilation [13].

## 3. Results

### 3.1. Microstructure of the Samples before Hot Compression

**Figure 1:**
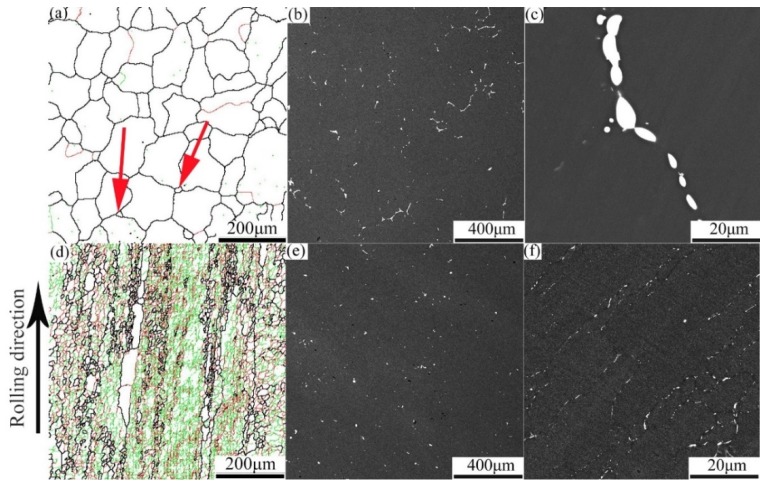
Microstructures of homogenized samples (**a**–**c**) and pre-rolled, T3 samples (**d**–**f**) before hot compression, showing boundaries based on EBSD with misorientation angles >15° as black lines, 2°–15° as red lines and <2° as green lines (**a**,**d**), and BSE images showing second phase particle characteristics (**b**,**c**,**e**,**f**) at different magnifications.

The homogenized microstructure before deformation shows equiaxed grains with an average grain size of 108 μm. Some new grains exist at the corners of the grain boundaries, as marked for example by the red arrows in Figure 1a. However, for the pre-rolled (T3) material, the average grain size is 21 μm, and large populations of both sub-grains (delineated by red lines) and recrystallized grains (black lines) are observed in Figure 1d.

The second phases are different in the two conditions as well. In the homogenized sample before deformation, the second phases are discontinuously distributed along the grain boundaries (Figure 1b) and some fine precipitates can be observed along the grain boundaries in Figure 1c. For the pre-rolled (T3) sample, small amounts of spherical and rod-like precipitates are present (Figure 1e,f), which are finer and more randomly distributed than in the homogenized material. Therefore, the major differences in the microstructure between the homogenized and pre-rolled (T3) samples are the grain size, the types of grains, the precipitate size and the number of precipitates.

### 3.2. Flow Stress Behavior

**Figure 2:**
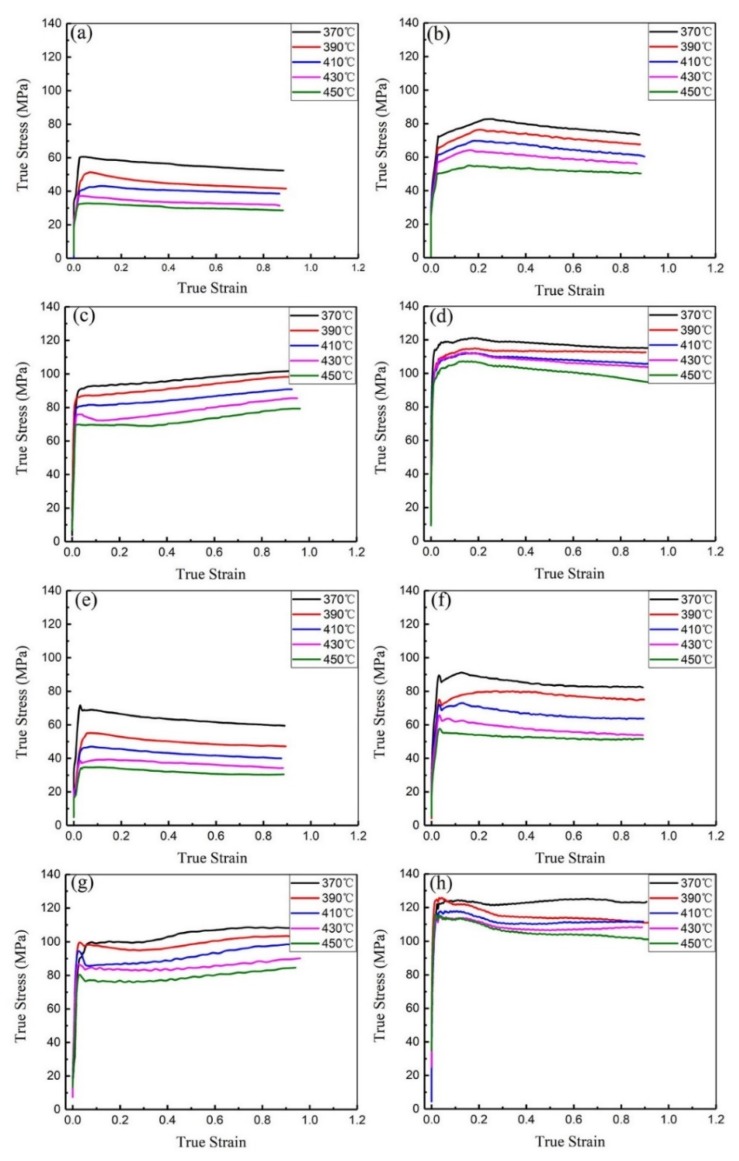
True stress–true strain curves of alloy 7055 during hot compression deformation after homogenization (**a**–**d**) and pre-rolled (T3) (**e**–**h**) at different strain rates: (**a**,**e**) $\dot{\varepsilon }$ = 0.01 s^−1^; (**b**,**f**) $\dot{\varepsilon }$ = 0.1 s^−1^; (**c**,**g**) $\dot{\varepsilon }$ = 1 s^−1^; (**d**,**h**) $\dot{\varepsilon }$ = 10 s^−1^.

Typical true stress-true strain curves obtained during hot compression are presented in Figure 2. In general, the flow stresses of homogenized samples (Figure 2a–d) are lower than those for the pre-rolled (T3) samples (Figure 2e–h) under the same compression conditions. This is consistent with the microstructural differences, that is, the flow stresses are lower with the larger grain sizes and coarser precipitates in the homogenized samples.

For both materials, the level of the flow stress consistently increases with decreasing deformation temperature. For each curve, the value of the true stress increases rapidly at the beginning of deformation and then remains unchanged or decreases slightly to some extent after attaining the peak stress. The increase of stress at the early stage of plastic deformation, observed in some samples, is caused by work hardening resulting from dislocation multiplication. After the peak, the stress may be constant due to a good balance between work hardening and dynamic softening, or the stress may decrease due to a predominant dynamic softening effect. For example, it seems that dynamic softening offsets work hardening after the true strain exceeds about 0.05 in Figure 2e. There are different mechanisms of dynamic softening, and at low strain rates, the apparent decline of stress in the higher temperature range is often a result of more recrystallization [14]. As a result, there is a significant decrease in true stress after a true strain of about 0.05 in Figure 2f–g, but this is much less evident in the results from the homogenized samples in Figure 2a–d.

In the pre-rolled (T3) condition, more sub-grains exist than in the homogenized condition, as shown in Figure 1a,d. When the pre-rolled (T3) sample is heated to the deformation temperature, the higher stored energy and the moving of sub-grains [15], favors dynamic softening.

**Figure 3:**
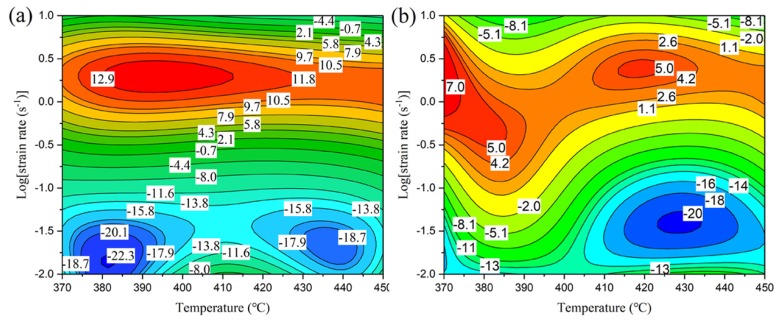
Decline ratio map of flow stress for alloy 7055 deformed after (**a**) homogenization and (**b**) pre-rolling (T3).

In order to describe the drop in flow stress during hot deformation at different deformation temperature and strain rates, a flow stress decline ratio map is introduced in Figure 3, based on the definition of R_d_ in Equation (1) [16]

(1) $$R_{d}\left(\%\right)=\frac{\sigma_{s}-\sigma_{p}}{\sigma_{p}}\times 100\%$$

where *σ_s_* is the value of flow stress at the end of the deformation (taken at a true strain of 0.8 for this analysis) and *σ_p_* is the peak flow stress.

From the flow stress decline ratio map, it is clear that the most significant decrease of flow stress (blue areas in Figure 3) happens mainly in regions with a low strain rate of 0.01 to 0.1 s^−1^ ($\log\dot{\varepsilon }$ = −2 to −1). At this strain rate level, the flow stress drops more for the homogenized condition than for the pre-rolled (T3) condition when the deformation temperature is in the range of 370 °C to 390 °C. The highest values of R_d_ (R_d_ > 0) for the homogenized condition occur in the temperature range of 370 °C to 450 °C at strain rates from 0.3 s^−1^ to 10 s^−1^ ($\log\dot{\varepsilon }$ = −0.5 to 1), while for the pre-rolled (T3) condition, the highest values appear at temperatures of 370 °C to 400 °C for strain rates from 0.1 s^−1^ to 6.3 s^−1^ ($\log\dot{\varepsilon }$ = −1 to 0.8) and 400 °C to 450 °C for strain rates from 0.1 s^−1^ to 5 s^−1^ ($\log\dot{\varepsilon }=$ −1 to 0.7). The regions with positive values of R_d_ are relatively smaller for the pre-rolled (T3) condition, which means that the stability of the flow stress in the pre-rolled (T3) condition is higher than in the homogenized condition for deformation at high temperatures. When the alloy is deformed at a higher strain rate or lower temperature, finer precipitates can reduce the nucleation energy of recrystallization and sub-grains can transform more easily into equiaxed grains [17,18]. This results in reduced stress concentrations at the corners of grain boundaries. However, the value of R_d_ (R_d_ > 0) for the homogenized condition is higher than for the pre-rolled (T3) condition when deformation is carried out under the same temperature and strain rate. It can be deduced that finer precipitates and sub-grains are helpful to resist non-uniform deformation during hot compression, especially at lower temperatures or higher strain rates.

Another interesting phenomenon is that single flow stress peaks and stress oscillations are observed in both homogenized and pre-rolled (T3) conditions during deformation, and the value of R_d_ is higher in homogenized than in the pre-rolled (T3) condition. Yamagata [19,20] reported that multiple peaks due to stress oscillation could be induced as a result of discontinuous dynamic recrystallization (DDRX) in pure aluminum. Changing values of R_d_ may therefore correspond to various deformation softening mechanisms, which will be discussed in the following sections.

### 3.3. Constitutive Equation and Processing Map

For a better understanding of the hot deformation behavior of alloy 7055, both constitutive equations and processing maps can be established on the basis of the experimental strain–stress curves. The constitutive equation is used to model the effects of deformation temperature and strain rate, which can be described by the Zener–Hollomon parameter Z, as [16,17]

(2) $$Z=\dot{\varepsilon }exp\left(\frac{Q}{RT}\right)$$

where $\dot{\varepsilon }$ represents the strain rate, *Q* is the activation energy of hot deformation, *R* is the universal gas constant, and *T* is the deformation temperature.

The value of *Q* can be obtained from the strain–stress curves using the expression [14]

(3) $$Q=Rn\frac{d\left\{ln\left[sinh\left(\alpha \sigma \right)\right]\right\}}{d\left(1/T\right)}$$

where α and *n* are material constants, and σ is the true stress.

Based on the equations above, the average values of activation energy for the homogenized and pre-rolled (T3) samples are 122.7 kJ/mol and 135.6 kJ/mol, respectively. The reason for the higher value of *Q* for the pre-rolled (T3) condition may be that there are more fine precipitates to hinder dislocation movement in this condition.

Table 1 shows the values of ln(Z) for the compression deformation of the homogenized and pre-rolled (T3) materials based on the experimentally derived *Q* values. At high ln(Z) values (above 20), the main deformation softening mechanism will change from dynamic recovery to dynamic recrystallization [21]. The deformation mechanism can be deduced from features on the strain–stress curves, such as stress increases, stress decreases and stress fluctuations. Flow stress fluctuations are typical for higher values of ln(Z) corresponding to strain rate ≥0.1 s^−1^ as illustrated in Figure 2. Multiple peaks are generally observed due to DDRX [16], so it is reasonable to deduce that higher values of ln(Z) are closely related to DDRX at higher strain rate and lower temperatures. During deformation at high strain rates, severe deformation heating helps in the formation of sub-grains via boundary migration rotation and developing high-angle grain boundaries [22]. In addition, lower deformation temperatures offer less energy for boundary migration as sub-grains rotate.

The higher decrement of true strain–stress at the beginning of deformation (decrease of flow stress at the true strain about 0.05) in the pre-rolled (T3) condition (Figure 2) can be explained by the difference in the microstructures before deformation. Sub-grains can easily transform into high angle grain boundaries, so more sub-grains grow into equiaxed grains (recrystallized grains) during compression of the pre-rolled (T3) sample than homogenized sample due to the existence of many more sub-grains. That also explains why the value of ln(Z) in the pre-rolled (T3) condition is higher than that for homogenized.

**Figure 4:**
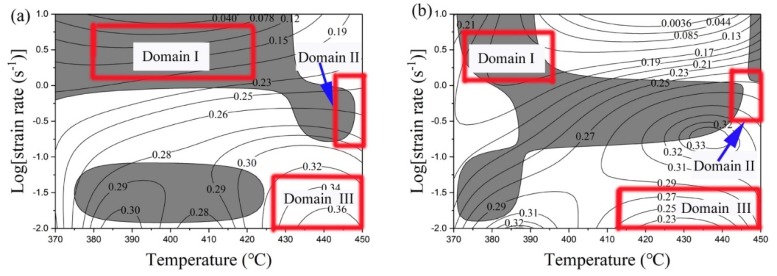
Processing maps for aluminum alloy 7055 at a true strain of 0.8 after (**a**): homogenization and (**b**): pre-rolling (T3). The domains are discussed in Section 3.2.

Processing maps were generated to determine the appropriate process domains. Figure 4 illustrates processing maps for a temperature range of 370 °C to 450 °C and a strain rate range of 0.01 s^−1^ to 10 s^−1^ ($\log\dot{\varepsilon }=$ −2 to 1) at a true strain of 0.8. The contour lines in the maps represent the value of power dissipation η, as defined in the following equation [23]

(4) $$\eta =\frac{2m}{m+1}$$

in which m can be expressed as

(5) $$m=\frac{\partial \left(\ln\sigma \right)}{\partial \left(\ln\dot{\varepsilon }\right)}$$

where σ is the flow stress at a strain value of 0.8, $\dot{\varepsilon }$ is strain rate.

For the identification of flow instabilities during hot deformation of materials, a dimensionless parameter, ξ, was introduced [23]

(6) $$\xi =\frac{\partial \ln\left[m/\left(m+1\right)\right]}{\partial \ln\dot{\varepsilon }}+m$$

The shaded areas in the maps indicate the unstable domains (ξ < 0) during thermal processing. It is observed that the main unstable domains for homogenized and pre-rolled (T3) samples during deformation are situated in a temperature range from 370 °C to 430 °C with strain rates of 1 s^−1^ to 10 s^−1^ ($\log\dot{\varepsilon }=0\;\mathrm{to}\;1$), and a temperature range from 370 °C to 450 °C with strain rates 0.1 s^−1^ to 1 s^−1^ ($\log\dot{\varepsilon }=-1$ to 0), respectively. The overlap between the unstable domain for homogenized and pre-rolled (T3) is the temperature range from 370 °C to 390 °C and strain rates from 1 s^−1^ to 10 s^−1^ ($\log\dot{\varepsilon }=0$ to 1). As mentioned above, severe deformation heating resulting from high strain rates offers more energy for boundary migration. Dynamically recrystallized regions at lower strains will tend to form around the old grains. With an increase of strain, the edges of those domains with high local stress resulting from the softening by new grains will promote recrystallization [24]. Thus, bands of recrystallized grains are formed and will merge and broaden to develop a shear zone. Shear zones are expected to generate cracks in order to relieve local stress concentrations when the alloy is deformed at higher strains, which is not desirable for process control.

Generally speaking, a region with high power dissipation corresponds to a processing window that is most suitable for hot deformation. There are three high power dissipation regions for the homogenized condition at low strain rates. As shown in Figure 4a, the first peak of power dissipation is about 30%, in the temperature range from 380 °C to 390 °C and at a strain rate of 0.01 s^−1^ ($\log\dot{\varepsilon }=-2$). The second peak of power dissipation is about 28%, in the deformation temperature range from 400 °C to 410 °C and at a strain rate of 0.01 s^−1^ ($\log\dot{\varepsilon }=-2$). The last region with the highest power dissipation efficiency of 36% is in the deformation temperature range from 430 °C to 450 °C and at a strain rate of up to 0.01 s^−1^ ($\log\dot{\varepsilon }=-2$), which is optimum for processing due to the wide temperature window.

Comparatively, for the pre-rolled (T3) condition, the most suitable processing domain is located in a deformation temperature range from 410 °C to 450 °C and at a strain rate ranging from 0.01 s^−1^ ($\log\dot{\varepsilon }=-2$) to 0.03 s^−1^ ($\log\dot{\varepsilon }=-1.5$), as shown in Figure 4b. Furthermore, suitable process windows can also be deduced from the flow stress decline ratio map shown in Figure 3. In this map, a lower decline ratio indicates more stress softening in the deformation stage, which is conducive to smooth processing. As a result, a domain with a lower decline ratio corresponds to the best hot workability.

A comparison between the decline map and processing map (Figure 3 and Figure 4) reveals that the optimum hot-working conditions for the homogenized material are in the temperature range of 405–450 °C and over a strain rate range of 0.01−0.08 s^−1^ ($\log\dot{\varepsilon }=-2\;\mathrm{to}-1.1$), while for the pre-rolled (T3) material, the most suitable processing temperature range is 430–450 °C with a strain rate range of 0.01 s^−1^ to 0.1 s^−1^ ($\log\dot{\varepsilon }=-2\;\mathrm{to}-1$).

### 3.4. Microstructure Evolution

**Figure 5:**
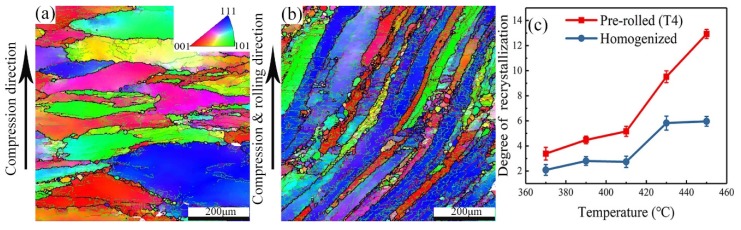
Inverse pole figure (IPF) maps of alloy 7055 after (**a**) homogenization and (**b**) pre-rolled (T3), followed by hot deformation at 450 °C with a strain rate of 1 s^−1^, with (**c**) showing the degree of recrystallization for both.

Figure 5 illustrates the deformed microstructures of the homogenized and the pre-rolled (T3) samples that were hot compressed at 450 °C with a strain rate of 1 s^−1^. It is evident from Figure 5a that for this homogenized and deformed sample, the compressed grains become flat and elongated in the radial direction. New grains, mainly distributed along the original grain boundaries in Figure 1a, have developed and grown (Figure 5a). According to the data acquired by EBSD, low-angle boundaries can be observed in the grain interiors. Similarly, the pre-rolled (T3) sample after hot deformation also shows low-angle boundaries in the grain interiors and new grains along the original grain boundaries (Figure 5b). One difference is that in the latter, some elongated grains along the rolling direction are bent. The elongated grains with narrow spacing, originating from hot rolling before solution treatment in the pre-rolled (T3) sample, were compressed with loads parallel to the rolling direction, which led to grain warping.

The degree of recrystallization shown in Figure 5c, based on statistics from Channel 5 software, increases with increasing temperature from 370 °C to 450 °C. When the alloy is deformed at a low deformation temperature, a high density of tangled dislocations is generated in the grain interior and then develops into dislocation walls [25]. The migration rate of dislocations is improved with increasing temperature, which leads to an easier occurrence of dynamic recrystallization. In addition, the degree of recrystallization in the homogenized sample is less than in the pre-rolled (T3) sample for any given deformation condition. It is well known that the driving force for recrystallization correlates positively to dislocation density but negatively to grain size [26]. The pre-rolled (T3) sample had been stretched to 2% after solution treatment, which induced more dislocations than in the homogenized sample. Furthermore, the pre-rolled (T3) sample also has a smaller average grain size than the homogenized one, as mentioned above (Figure 1). Therefore, the driving force for recrystallization for the pre-rolled (T3) sample with a smaller grain size and more dislocations is higher than for the homogenized sample, which accounts for the larger degree of recrystallization during hot deformation in Figure 5c.

**Figure 6:**
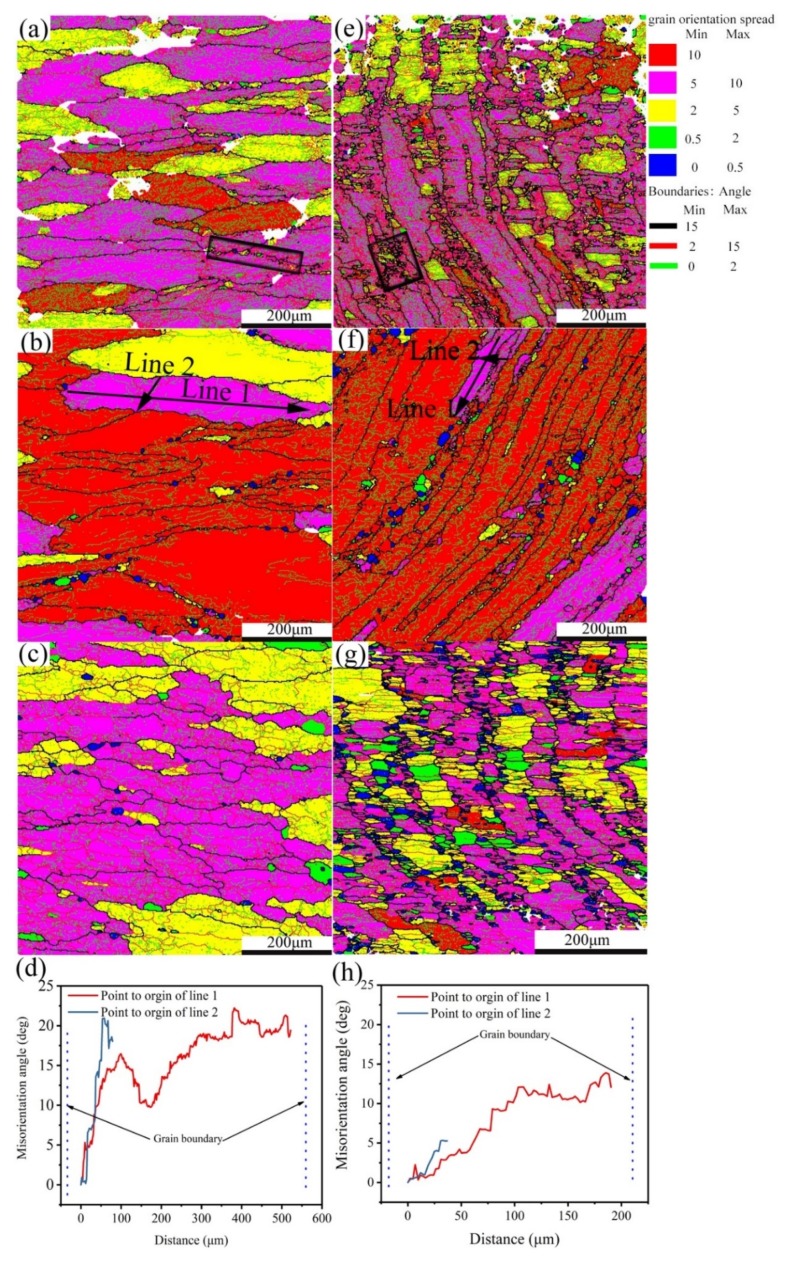
Grain orientation spread (GOS) maps for alloy 7055 after (**a**–**d**) homogenization and (**e**–**h**) pre-rolling (T3), followed by deformation in the unstable domain (**a**–**e**) of 390 °C with a strain rate of 1 s^−1^, deformation near the unstable domain (**b**,**f**) of 450 °C with a strain rate of 1 s^−1^, and deformation in the optimum processing domain (**c**,**g**) of 430 °C with a strain rate of 0.01 s^−1^; (**d**,**h**) correspond to the misorientation profiles of lines 1 and 2 in (**b**,**f**), respectively.

According to the processing maps illustrated in Figure 4, three representative domains can be chosen to study the microstructure evolution, i.e., domain I is an unstable region, domain II is all areas close to the unstable domain boundary (with typical areas pointed out by the blue arrows), and domain III represents the optimum processing domain. Grain orientation spread (GOS) maps of homogenized or pre-rolled (T3) samples after deformation in those domains are shown in Figure 6. Shear bands with fine equiaxed grains [27] are identified by black rectangles in Figure 6a,e. GOS is the average deviation in orientation between each point in a grain and the average orientation of the grain [28,29]. A low GOS value always means low plastic deformation and uniform misorientation, which can be used to identify the recrystallized grain (below 2°). From Figure 6a–c,e–g, it can be seen that the recrystallized grains are surrounded by deformed grain boundaries or located at the triple junctions of deformed grains in both samples. The number of recrystallized grains (misorientation angle less than 2°, shown in green and blue in Figure 6 decreases following the sequence from optimum processing domain to near unstable domain to unstable domain. The optimum processing domain with its low value of ln(Z) is easy to recrystallize due to more energy and time for dislocation movement.

**Figure 7:**
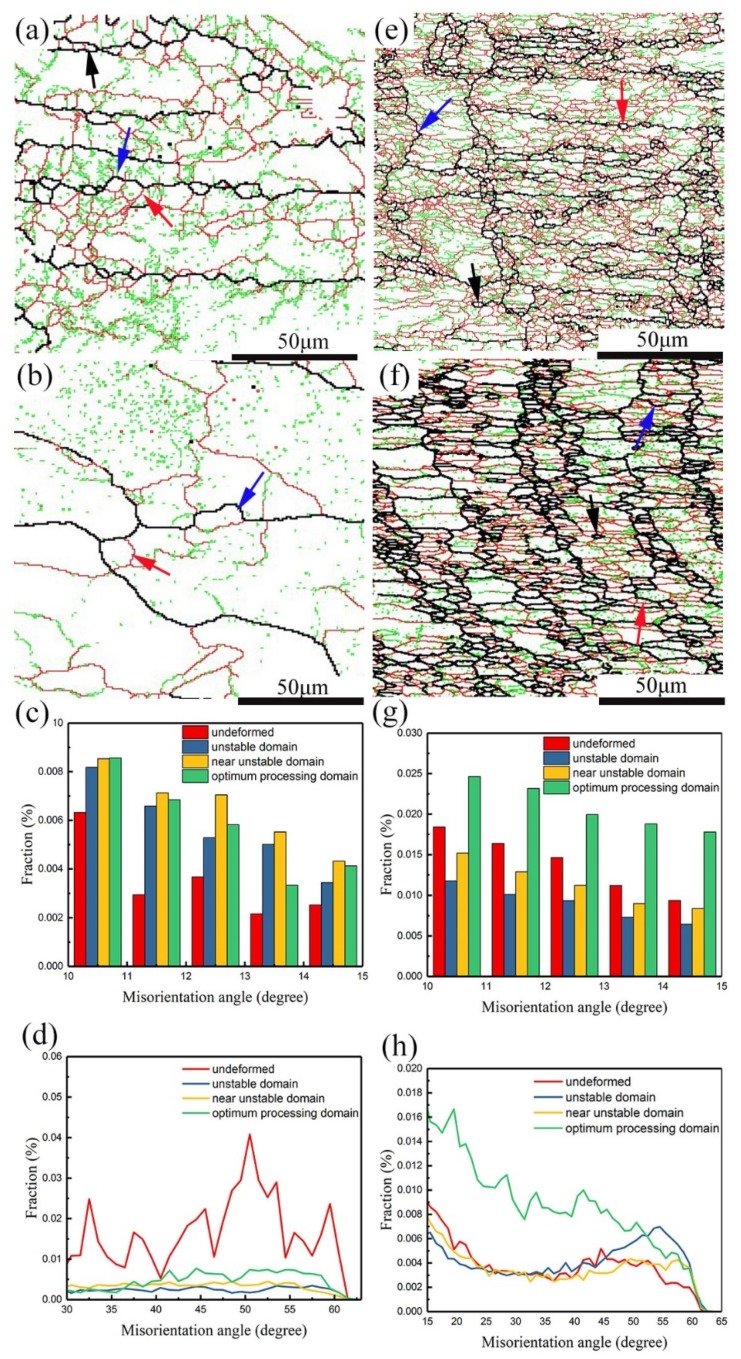
Grain boundary maps for homogenized (**a**,**b**) and pre-rolled (T3) (**e**,**f**) conditions, and grain boundary orientations for homogenized (**c**,**d**) and pre-rolled (T3) (**g**,**h**) conditions, where (**a**,**e**) are in unstable domains, and (**b**,**f**) are in optimum processing domains.

Through the misorientation profile analysis performed on line 1 and line 2 in Figure 6b,f of homogenized and pre-rolled (T3) samples after deformation, the point-to-origin (cumulative) misorientation in the grains of the homogenized and deformed alloy was over 15°, indicating a good development of the progressive sub-grain rotation, which is a sign of continuous dynamic recrystallization (CDRX) [30,31]. Meanwhile, the increasing fraction of misorientation from 10° to 15° also suggests the occurrence of CDRX [31], which can be found in Figure 7c. However, the reduced amount of misorientation within 10–15° and the low point-to-origin misorientation value after deformation of the pre-rolled (T3) condition means less grains were formed by progressive sub-grain rotation due to the existence of sub-grains before deformation. When CDRX occurs, the flow stress curve generally attains a peak stress and then shows very little softening upon further strain [16,22]. The fact that the grains are not fully surrounded by high-angle grain boundaries indicates that the CDRX transformation and formation of new grains are incomplete [21]. Grains with such CDRX features that are comprised of either high-angle grain boundaries (HAGB) or mid-angle grain boundaries (MAGB) with an angle range of 2° to 15°, are identified by red arrows in Figure 7a,e. Similarly, in the pre-rolled (T3) condition, grains consisting of HAGB and LAGB are also present and are identified by red arrows in Figure 7e–f.

Grain boundary bulging (BLG), resulting from grain boundaries being pinned by impurities/precipitates or sub-grains [31,32], has been found in the homogenized and deformed samples (Figure 7a,e, blue arrow). BLG, as a mechanism of (DDRX), has been widely acknowledged [32,33] and always leads to fluctuation of flow stress in the stress–strain curves [22]. In this case, the precipitate-pinned grain boundary resulted from dynamic precipitation, where fine precipitates were dynamic precipitated during hot compression [34]. However, for the pre-rolled (T3) and deformed samples, the grain boundary is pinned by large number of sub-grains (marked by red and green lines shown in Figure 1d). The microstructure after DDRX is the so-called “necklace” structure [33,35], which appears in the grain boundaries shown in Figure 7a. Similar but finer “necklace” structures appear in the pre-rolled (T3) and deformed sample due to its smaller grain size in the original structure before deformation (Figure 1).

In addition, an interesting characteristic is that some small grains consisting of original HAGB and MAGB are located at the corners of deformed grains, as highlighted by the black arrow in Figure 7a. Those flat and thin grains coincide with the features of geometric dynamic recrystallization (GDRX). When the strain reaches a critical level and the grain thickness reaches twice the sub-grain diameter, the concave serrations of the original grain boundaries will contact each other and perforate the original grain. This mechanism of GDRX was proposed by McQueen [35,36]. In the pre-rolled (T3) and deformed condition, the structure of GDRX shown in Figure 7a is not observed. However, other distinctive grains (identified by the black arrows in Figure 7e–f) with grain boundary misorientation angles above 30° are found. Those grains are not growing from recrystallized nuclei but are originating from the original sub-grains. This type of recrystallization mechanism with ‘interrupt’ characteristics is known as meta-dynamic recrystallization (MDRX) [37,38,39]. The grain boundary orientation plot in Figure 7h indicates that the fraction of grain boundary orientations above 30° is generally increased after deformation, especially in the domain of optimum processing. There is an inconsistent tendency for misorientations within 10–15° in the two types of samples, except in the optimum processing domain. When the pre-rolled (T3) sample is deformed at higher strain rates, a large number of LAGBs grow into HAGBs and some LAGBs are generated in the recrystallized grains, which causes a lower fraction of 10–15° misorientations after deformation compared to the undeformed condition.

As discussed above, CDRX and DDRX both occur in samples after homogenization or pre-rolling (T3) followed by deformation, but GDRX only occurs in the homogenized and deformed condition and MDRX only occurs in the pre-rolled (T3) and deformed condition. In order to investigate the proportion of each mechanism in each condition, the grains were divided into different categories based on their microscopic characteristics and misorientation. The statistical results are listed in Table 2, where DDRX refers to grains exhibiting the BLG feature, CDRX refers to grains without BLG and not fully comprised of high angle grain boundaries, GDRX refers to grains defined by their morphology as flat and thin or with a thickness reaching twice the sub-grain diameter, and MDRX refers to grain boundaries with misorientations above 30°. It is obvious that DDRX as the main mechanism tends to appear in the unstable domain, but CDRX is inclined to occur in the optimum processing domain. Combined with Figure 3, it is evident that R_d_ values of less than −15 in the temperature range from 410 °C to 450 °C and strain rate range from 0.01 s^−1^ to 0.1 s^−1^ ($\log\dot{\varepsilon }=-2\;\mathrm{to}-1$) result from CDRX in both conditions. In addition, DDRX mainly appears in the unstable and near-unstable domains, which are located in the region with R_d_ above zero (Figure 3 and Figure 4). GDRX only exists in the optimum processing domain for the homogenized and deformed sample due to less distortion at lower strains and a higher deformation temperature. Inversely, it is easy to observe MDRX grains in the pre-rolled (T3) and deformed condition at lower values of ln(Z) because of interrupted conditions (cooling and natural aging after hot rolling) for grain growth before deformation.

## 4. Conclusions

Samples of aluminum alloy 7055 in different heat treatment states were compressed in a temperature range of 370–450 °C at strain rates of 0.01–10 s^−1^. Two heat treatments were considered: (i) homogenized after casting, and (ii) pre-rolled (T3) i.e., naturally aged after casting, homogenization, rolling, solution treatment and stretching. The flow stress curves and microstructure evolution were investigated. Some conclusions are drawn as follows:

1. The flow stress of both the pre-rolled (T3) and homogenized samples during hot deformation increases with decreasing temperature and increasing strain rate as expected, but for given compression conditions, the flow stress of the pre-rolled (T3) samples is higher than for the homogenized ones.
2. The hot deformation activation energy, calculated on basis of flow stress, is 122.7 kJ/mol and 135.6 kJ/mol after homogenization and pre-rolling (T3), respectively.
3. The degree of recrystallization in both the homogenized and pre-rolled (T3) conditions increased with increasing deformation temperature. For given compression conditions, higher fractions of recrystallization were observed in the deformed samples after pre-rolling (T3) than after homogenization due to finer second phase particles, smaller grains, and numerous sub-grains.
4. Discontinuous dynamic recrystallization (DDRX) and continuous dynamic recrystallization (CDRX) were identified as the main softening mechanisms for both the homogenized and the pre-rolled (T3) conditions during deformation. Geometric dynamic recrystallization (GDRX) is only observed in the homogenized condition and meta-dynamic recrystallization (MDRX) is only observed in the pre-rolled (T3) condition during deformation. Changes in the decline ratio of flow stress R_d_ correspond to the various softening mechanisms.

## Figures and Tables

**Table 1:** Ln(Z) at a true strain of 0.8 for various deformation conditions.

Temperature	ln(Z) for the Homogenized and Deformed Condition				ln(Z) for the Pre-rolled (T3) and Deformed Condition			
	0.01 s^−1^	0.1 s^−1^	1 s^−1^	10 s^−1^	0.01 s^−1^	0.1 s^−1^	1 s^−1^	10 s^−1^
370 °C	15.941	18.244	20.546	22.849	20.746	23.048	25.351	27.653
390 °C	15.321	17.624	19.927	22.229	19.981	22.284	24.586	26.889
410 °C	14.738	17.041	19.343	21.646	19.261	21.564	23.867	26.169
430 °C	14.188	16.491	18.793	21.096	18.582	20.885	23.188	25.490
450 °C	13.668	15.971	18.273	20.576	17.941	20.244	22.546	24.849

**Table 2:** Proportion of each mechanism operative in deformed samples after homogenization or pre-rolling (T3).

Recrystallization Type	Homogenization			Pre-rolling (T3)		
	Unstable Domain	Near Unstable Domain	Optimum Processing Domain	Unstable Domain	Near Unstable Domain	Optimum Processing Domain
DDRX	75%	56%	9%	66%	28%	28%
CDRX	18%	31%	91%	23%	38%	40%
GDRX	7%	13%	-	-	-	-
MDRX	-	-	-	19%	34%	32%
